# Vehicle Classification Using the Discrete Fourier Transform with Traffic Inductive Sensors

**DOI:** 10.3390/s151027201

**Published:** 2015-10-26

**Authors:** José J. Lamas-Seco, Paula M. Castro, Adriana Dapena, Francisco J. Vazquez-Araujo

**Affiliations:** Grupo de Tecnoloxía Electrónica e Comunicacións (GTEC), Departamento de Electrónica e Sistemas, Facultade de Informática, Universidade da Coruña, Campus da Coruña, 15071 A Coruña, Spain; E-Mails: lamas@udc.es (J.J.L.-S.); adriana.dapena@udc.es (A.D.); fjvazquez@udc.es (F.J.V.-A.)

**Keywords:** analytical methods, data acquisition, inductive loop detectors, intelligent transportation systems, sensor applications, sensor devices, sensor modeling, signal processing, software for sensors, traffic applications

## Abstract

Inductive Loop Detectors (ILDs) are the most commonly used sensors in traffic management systems. This paper shows that some spectral features extracted from the Fourier Transform (FT) of inductive signatures do not depend on the vehicle speed. Such a property is used to propose a novel method for vehicle classification based on only one signature acquired from a sensor single-loop, in contrast to standard methods using two sensor loops. Our proposal will be evaluated by means of real inductive signatures captured with our hardware prototype.

## 1. Introduction

Since its introduction in the 1960s, Inductive Loop Detectors (ILDs) are the most commonly active sensors used in traffic management systems [1,2,3,4,5,6,7,8]. These sensor systems mostly need accuracy and reliability when estimating vehicle speed with a minimum delay in control strategies. The estimate obtained from dual loop detectors is usually accurate [9,10,11,12], but it requires a proper maintenance of both loops, which implies that it is not the optimum solution in terms of cost. Moreover, only one loop is available in most of traffic systems. Although some algorithms have already been developed for single-loop classification [13,14,15] and single-loop speed estimation [16,17], how to achieve enough accuracy using only one loop is still an open question.

In this work, we will present a method for vehicle identification based on analyzing the inductive signatures in the frequency domain instead of working in the time domain. The proposed descriptor in the transform domain will be used for vehicle classification by means of a simple threshold-based method.

We will show some experimental results obtained with two different methods. The first set of experiments has been performed using a powerful testing tool developed by us based on [18]. Its main advantage is that it provides us useful prior information before the actual testing in a real scenario, thus reducing the necessary time and resources. The second set of experiments uses a hardware prototype capable of obtaining simultaneous inductive signatures of vehicles traveling on a road with minimal cost. Based on Time-Division Multiplexing (TDM) with multiple oscillators, one for each inductive loop, the system detects the presence of vehicles by means of a shift in the oscillation period of the selected loop and registers the signature of the detected vehicles.

This paper is organized as follows. In Section 2, we show the proposed method for feature extraction from inductive signatures based on a descriptor obtained by means of a spectral analysis. Our method is introduced as opposed to the standard and well-known strategy based on the vehicle length estimate. Section 3 shows the experiments developed in this work, where a novel threshold-based method for vehicle classification using the proposed tool is presented. The results will be analyzed in Section 4. Finally, Section 5 is devoted to the concluding remarks.

## 2. Proposed Method

ILDs are usually employed in traffic management systems to estimate vehicle parameters such as speed and length. In this work, we study ILDs based on period shift. These ILDs use a reference clock signal whose frequency is of several MHz, typically between 20 and 1000 times greater than the oscillation frequency of the inductive loop we are employing for measurement. The period of the oscillation signal is calculated as the number of cycles *N* of the reference clock signal in $m_{c}$ cycles of the oscillation signal. When a vehicle stops or passes over the loop the oscillation frequency increases, so the period (and thus the number of cycles *N*) decreases. The pulses from the oscillation loop are carried to a counter input, so that when a fixed number of pulses $m_{c}$ is reached, the measured value *N* is captured from a timer working at the frequency $f_{r}$ of the reference clock signal. The amplitude of the signatures is determined by means of the difference between the measured value *N*, obtained every 10 ms, and that obtained at rest, which is calculated and registered by the measurement equipment. This value is referred to as ΔN, so the oscillation period shift is given by $\Delta T=\Delta N/m_{c}$. This parameter ΔT gives us the amplitude of the vehicle inductive signature at a time instant.

**Figure 1:**
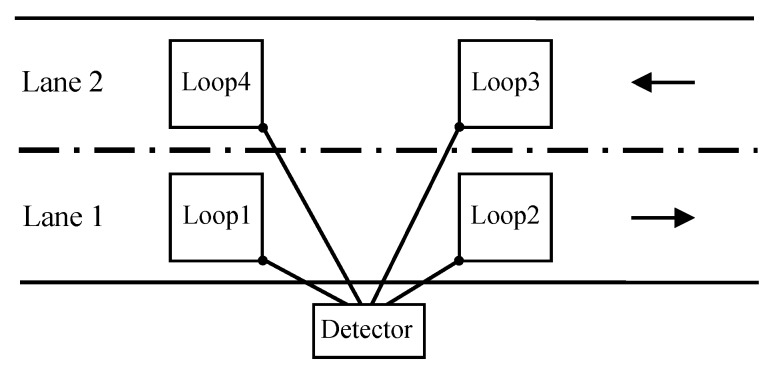
Interconnections from the inductive loop to the detector.

Figure 1 shows the typical system employed to capture vehicle signatures with two inductive loop sensors per road lane. It is apparent that, using such a configuration, two signatures are registered per vehicle. These sensors are squares with a side length denoted by *w* and a distance *d* between their centers. Let $r^{l}\left(t\right)$ be the inductive signature captured in loop *l*, with l=1,2 in lane 1 and l=3,4 in lane 2. These inductive signatures are composed of the sequence of ΔT values obtained by the method described previously. Let $t_{i}^{l}$ and $t_{f}^{l}$ be the initial and final time instants, respectively, of a vehicle passing over the loop. The standard methods for vehicle classification are based on estimating both the speed and length of the vehicle using the following expressions [19], accordingly, to the aforementioned notation

(1) $$\begin{array}{cc} \hat{s} & =\frac{d}{t_{i}^{2}-t_{i}^{1}} \end{array}$$

(2) $$\begin{array}{cc} \hat{L} & =\hat{s}\times \frac{\left(t_{f}^{1}-t_{i}^{1}\right)+\left(t_{f}^{2}-t_{i}^{2}\right)}{2}-w \end{array}$$

Conversely, in this paper we propose to compute the *L*-point Discrete Fourier Transform (DFT) of the *M* samples of $r^{l}\left(t\right)$ obtained between the time instants $t_{i}^{l}$ and $t_{f}^{l}$. Therefore, we will obtain the frequency domain signature

(3) $$\begin{array}{c} r^{l}\left[\omega_{k}\right]=\sum_{m=0}^{M-1}r^{l}\left(m\right)e^{-j\omega_{k}m},\;\;k=0,...,L-1 \end{array}$$

where $\omega_{k}=2\pi k/L$ denotes the frequency bin. Throughout this paper, we will always assume that the number of frequency bins *L* is greater than the window size *M*. After normalizing the absolute values by the first coefficient at k=0, *i.e.*, $\omega_{0}$, we get the normalized DFT

(4) $$\begin{array}{c} R^{l}\left[\omega_{k}\right]=\frac{|r^{l}\left[\omega_{k}\right]|}{|r^{l}\left[\omega_{0}\right]|} \end{array}$$

Considering $\omega_{k}>0$, we obtain the descriptor parameter proposed in this paper by finding the first local maximum of $R^{l}\left[\omega_{k}\right]$. The frequency bin of such peak will be denoted by *n*, and thus the descriptor that will be used in the classification of the vehicle is given by $R^{l}\left[\omega_{n}\right]$. As we demonstrate in Appendix B, this parameter is independent of the speed of the vehicle and of its lateral displacement over the inductive loop. In addition, in Section 4, we show that the descriptor will provide us with enough information to perform the classification of the vehicles with high accuracy.

## 3. Experimental Section

We are interested in assessing the vehicle classification capacity of the descriptor $R^{l}\left[\omega_{n}\right]$. For this purpose, we have developed two experiments: the first one, considering the inductive loop model described in [18] and the second one, using real acquired signatures. The application of the proposed inductive sensor in combination with the descriptors already described in Section 2 for vehicle classification will be evaluated by means of the third experiment also included in this section.

### 3.1. Experiment with an Inductive Loop Model

Firstly, we develop an inductive sensor model that provides us with a tool for testing without the need for actual on-site measurements.

Considering the resonant parallel circuit with equivalent inductance $L_{eq}\left(t\right)$ and capacity $C_{T}$ (Appendix A shows a detailed description of the equivalent circuit model), the shift in the oscillation period (which gives us the inductive signature) is determined as follows

(5) $$\Delta T=2\pi \left(\sqrt{L_{1}C_{T}}-\sqrt{L_{eq}\left(t\right)C_{T}}\right)$$

where

(6) $$L_{eq}\left(t\right)=\frac{L_{1}L_{2}\left(t\right)-M{\left(t\right)}^{2}}{L_{2}\left(t\right)}$$

In both expressions, $L_{1}$ is the inductance of the road loop, $L_{2}\left(t\right)$ is the vehicle inductance and M(t) is the mutual inductance. Note that $L_{2}\left(t\right)$ and M(t) depend on the position of the vehicle over the road loop and therefore, on the time instant *t*. The vehicle speed gives us different values of $L_{2}\left(t\right)$ and M(t) at a certain time instant *t*.

**Figure 2:**
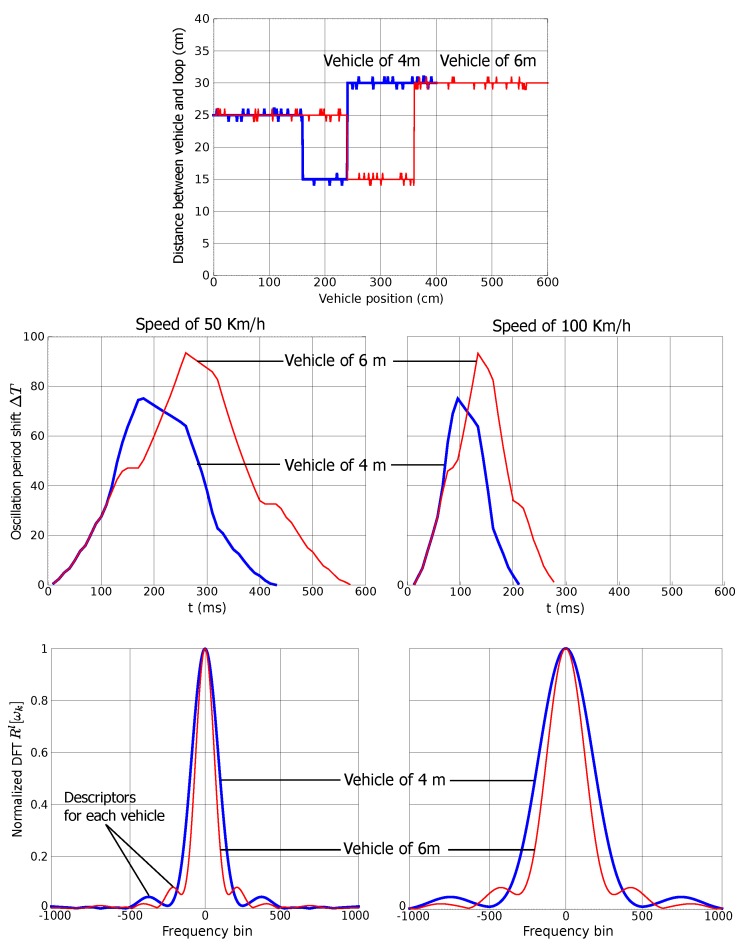
Software simulator: Examples of profiles (**Top**), signatures (**Middle**) and normalized DFT (**Bottom**) for two vehicles of 4 m and 6 m in length and for different speeds.

Figure 2 shows an example of the results obtained for vehicles of 4 m and 6 m in length with this model. The top figure shows the simulated vehicle profile, which represents the distance between the vehicle undercarriage and the road loop. The figure also represents the signatures obtained for vehicle speeds of 50 km/h and 100 km/h. It is interesting to note that the signature shape depends on the vehicle length, and that the signature length depends on the vehicle speed, but the shape remains mostly constant [2]. The bottom figure shows the DFT of the aforementioned simulated signatures with L=4096, although only the DFT central part is shown in the figure. Obviously, the vehicle speed has an influence on the DFT length, but the descriptor $R^{l}\left[\omega_{n}\right]$ is invariant to speed changes since the magnitude scaling is eliminated by the normalization in the frequency domain (see Appendix B). It is also significant to note that the values of the proposed descriptor are greater for vehicles of 6 m in length than for those of 4 m.

**Figure 3:**
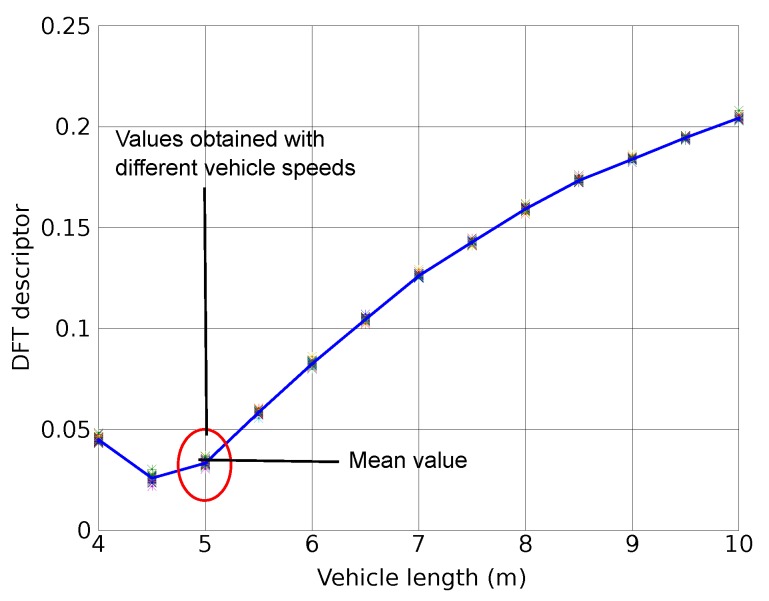
Software simulator: Signature descriptor for vehicle length from 4 m to 10 m, and speed from 20 km/h to 120 km/h.

Figure 3 shows the value of that descriptor $R^{l}\left[\omega_{n}\right]$ when the vehicle length is varying from 4 m to 10 m, and also the speed from 20 km/h to 120 km/h. The figure shows the obtained values and their corresponding mean value given the vehicle length. We can observe that their length produces significant changes in $R^{l}\left[\omega_{n}\right]$. On the other hand, the impact of speed changes is relatively small if the length remains unchanged. The figure shows a clear relationship between length and $R^{l}\left[\omega_{n}\right]$. The anomaly between 4 and 4.5 m is caused by the choice of vehicle undercarriage profile (shown in Figure 2) and the model employed for the simulation [18]. In our simulations we have observed that different vehicle profiles can cause different anomalies in the low length region, but in all cases the relationship between the DFT descriptor and the vehicle length remains significant.

**Figure 4:**
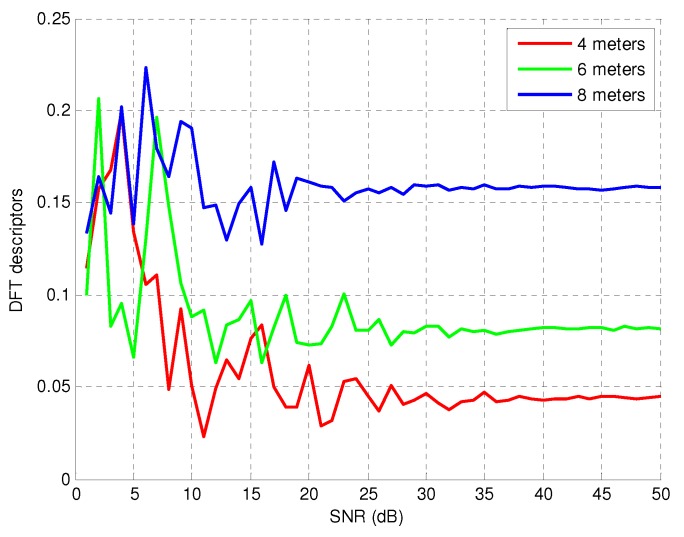
Impact of additive white Gaussian noise on the DFT descriptor.

Finally, in order to test the robustness of the descriptor against noise, we perform tests adding white Gaussian noise to the inductive signature signal. The impact of the Additive White Gaussian Noise (AWGN) on the DFT descriptor is shown in Figure 4. The noise is applied to inductive signatures obtained from three vehicles of 4 m, 6 m and 8 m in length using the vehicle profile depicted at the top of Figure 2. As can be seen in the figure, no significant effects on the DFT descriptor can be observed for Signal-to-Noise Ratios (SNRs) greater than 20 dB.

### 3.2. Experiment Using Real Acquired Signatures

**Figure 5:**
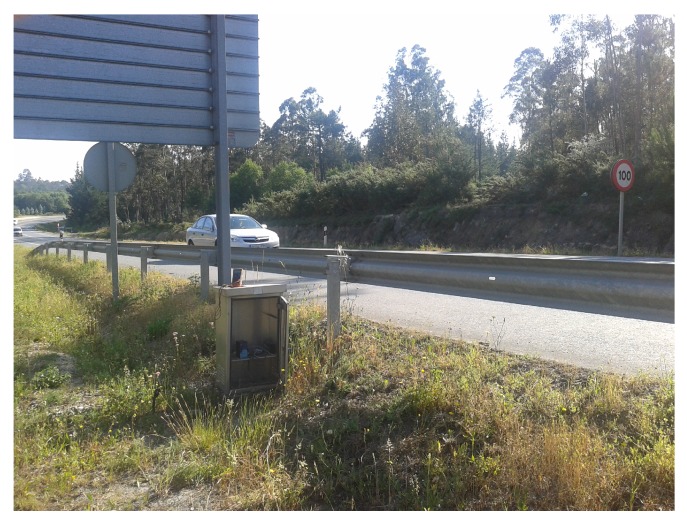
A photo of the measurement location in the AC-523 road (Ledoño-Meirama, kilometer 7, Spain), with GPS coordinates: 43.235941 (Lat.); −8.464462 (Long.).

In order to confirm the validity of the proposed classification method we have performed an experiment using a hardware implementation of an ILD [19]. This simulation will allow us to determine whether the actual profile of the car and its mass distribution affect the classification using the DFT descriptor. For the experiment we captured more than one thousand inductive signatures in two real scenarios: In the AC-523 road (Ledoño-Meirama, Spain), and in the AC-415 road (Pastoriza-Arteixo, Spain). A picture of the first location is shown in Figure 5. The detector equipment was located inside the cabinet of the Río Anllóns station, also shown in the photo. Both scenarios present the configuration shown in Figure 1 with square loop sensors with a side length of w=2m and a distance between their centers of d=5m.

The vehicles passing on the road will be classified using a threshold-based criterion. In the following, we will show the results obtained when those vehicles are classified according to three types: Cars, vans and trucks. We have used the aforementioned threshold-based criterion as follows

$$\begin{array}{cccccc} \hat{p}\leq \epsilon_{1} & \to \text{Car}; & & \epsilon_{1}<\hat{p}\leq \epsilon_{2}\to \text{Van}; & & \hat{p}>\epsilon_{2}\to \text{Truck} \end{array}$$

where $\hat{p}$ is the descriptor to be considered according to the criterion. This criterion can be employed for both length-based classification and for DFT descriptor-based classification, since there is a direct relationship between them. For the length-based classification, we will use the result of Equation (2). In the case of DFT-based classification, we will employ $R^{l}\left[\omega_{n}\right]$. The two prefixed thresholds, $\epsilon_{1}$ and $\epsilon_{2}$, are obtained empirically from a training stage.

At the same place where the inductive signatures were captured, we placed a video camera for the recording of the passing vehicles. Using the signatures and the video, an expert has classified all the signatures into the three different types considered in this work (cars, vans and trucks). Note that although in our work this process is manually performed by the expert, computer vision techniques could be used for the task [20,21]. In the AC-523 road, we have a total of 909 vehicles: 680 cars, 61 vans and 168 trucks. In AC-415 road, we have registered a total number of 1180 vehicles: 1022 cars, 79 vans and 79 trucks.

**Figure 6:**
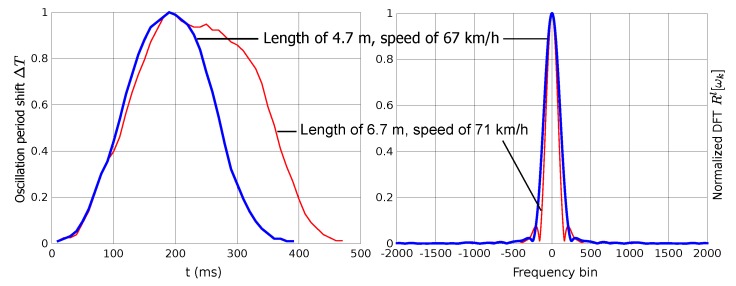
Experimental results: Examples of captured signatures.

Figure 6 shows two real signatures as an example, which correspond to a car and a van. The sampling interval is 10 ms. Since two signatures have been acquired for each vehicle, we have estimated speed and length using Equations (1) and (2). The car is 4.7 m in length passing with a speed of 67 km/h. The van is 6.7 m in length passing with a speed of 71 km/h. Similarly to the simulated signatures plotted in Figure 2, the largest signature produces the highest value of our descriptor $R^{l}\left[\omega_{n}\right]$.

**Figure 7:**
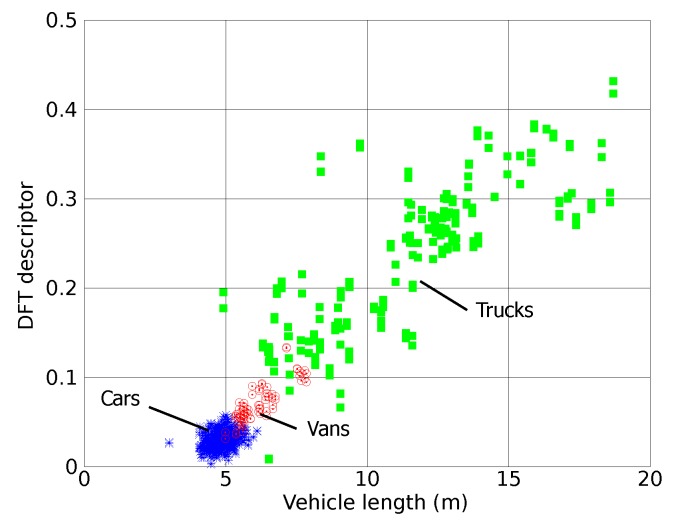
Experimental results: Signature descriptor compute from adquired signatures.

Considering the signatures acquired in the loop 1 of the AC-523 road, the descriptor values $R^{1}\left[\omega_{k}\right]$ given by the expression of Equation (4) with the vehicle length obtained as detailed in Equation (2) are computed. Figure 7 plots the values obtained in this way.

### 3.3. Experiment for Vehicle Classification

**Figure 8:**
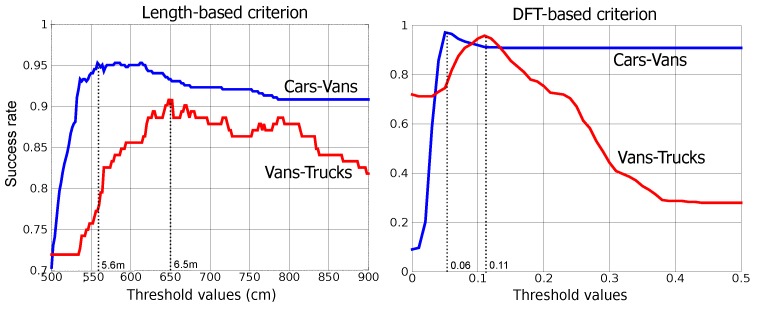
Experimental results: Sucess rates for different threshold values.

In the third experiment, we will obtain some results when the descriptors aforementioned are used for applications of vehicle classification. In the training stage, we have considered the signatures captured in the AC-523 road. All the loops have been employed to compute both length and speed, and only the loops 1 and 3 have been used for calculating the DFT descriptor. The value corresponding to the threshold $\epsilon_{1}$ is obtained when only cars and vans are considered, while the threshold value $\epsilon_{2}$ is obtained when only vans and trucks are computed. Figure 8 shows the success rate in vehicle classification, *i.e.*, the fraction or percentage of success in classifying a real car, van or truck passing on the road as car, van or truck, respectively, obtained for different threshold values. From this figure we conclude that the optimum values for those thresholds are $\epsilon_{1}=5.6$ and $\epsilon_{2}=6.5$ if the length-based method is used, and $\epsilon_{1}=0.06$ and $\epsilon_{2}=0.11$ if the DFT-based method is applied instead.

For the evaluation of the performance of both classification methods, we have considered the two experimental scenarios, *i.e.*, AC-523 and AC-415 roads. Table 1 shows the confusion matrices for each type of vehicle obtained in the AC-523 road, from the training phase (using the loops 1 and 3), and from considering the loops 2 and 4. Table 2 shows the results corresponding to the AC-415 road.

**Table 1:** Confusion matrices for AC-523 road.

	Length				DFT (Loops 1 and 3)				DFT (Loops 2 and 4)				
	Car	Van	Truck	%	Car	Van	Truck	%	Car	Van	Truck	%	Total
Car	666	14	0	**97.94**	669	11	0	**98.38**	666	14	0	**97.94**	680
Van	13	27	21	**44.26**	12	42	7	**68.85**	16	41	4	**67.21**	61
Truck	2	5	161	**95.83**	1	7	160	**95.24**	1	13	154	**91.67**	168
Total	681	46	182	**93.95**	682	60	167	**95.82**	683	68	158	**94.72**	909

**Table 2:** Confusion matrices for AC-415 road.

	Length				DFT (Loops 1 and 3)				DFT (Loops 2 and 4)				
	**Car**	**Van**	**Truck**	**%**	**Car**	**Van**	**Truck**	**%**	**Car**	**Van**	**Truck**	**%**	Total
Car	1013	7	2	**99.12**	998	22	2	**97.65**	1013	6	3	**99.12**	1022
Van	30	33	16	**41.77**	11	64	4	**81.01**	15	61	3	**77.22**	79
Truck	3	14	62	**78.48**	0	17	62	**78.48**	0	15	64	**81.01**	79
Total	1046	54	80	**93.90**	1009	103	68	**95.25**	1028	82	70	**96.44**	1180

## 4. Results and Discussion

The results in Figure 3, obtained using the software simulator, show that the proposed frequency domain descriptor depends on the vehicle length but not on their speed. These results have been confirmed with those obtained from real signatures measured with our sensor prototype, as it can be seen in Figure 7. This figure also shows that some van signatures present a frequency domain descriptor similar to those corresponding to car or truck signatures, which makes the task of identification of this type of vehicles quite difficult.

The results obtained for vehicle classification are shown in Table 1 (AC-523 road) and Table 2 (AC-415 road) in terms of the confusion matrices. Note that the diagonal entries correspond to correct identifications and that the off-diagonal elements of the confusion matrix correspond to crossed classifications or errors. The smallest number of errors is obtained when the proposed DFT-based criterion is applied, compared to the length-based one. Since car and van signatures are very similar, most classification errors are produced for vans but note that the DFT-based criterion proposed in this paper leads to lower error percentages in such case than those corresponding to the length-based criterion.

Finally, we compare the results obtained by our system with previous single-loop methods in the literature. Table 3 shows the different success rates extrapolated from the confusion matrices of those works when classifying cars, vans and trucks and applied to our vehicle distributions. Although the real scenarios are not the same and the vehicles are different, if we compare the results obtained from all the tests, the performance of our proposal is similar or better, with the additional advantage of using a very simple method. Note that although more than three vehicle types have been considered in some of those works, we restrict ourselves to the classification results achieved for cars, vans, and trucks.

**Table 3:** Comparison with other related literature works in terms of success rates.

Road	Oh *et al.* [2]	Ki and Bai [13]	Meta and Cinsdikici [14]	DFT Loops 1 and 3	DFT Loops 2 and 4
AC-523	88.45%	94.17%	95.05%	95.82%	94.72%
AC-415	88.98%	95.34%	95.68%	95.25%	96,44%

## 5. Conclusions

This paper shows that the DFT is an adequate tool to classify vehicles from inductive signatures because of the spectral features extracted from the frequency domain analysis. This analysis exhibits interesting properties. Firstly, it can be used with only one loop, as it is shown in the results section. Secondly, the DFT has been demonstrated to be independent to variations in the vehicle speed. Moreover, the experimental results performed with real signatures captured with our inductive sensor prototype have shown that the proposed DFT-based criterion obtains a significant reduction of the total error percentage when compared with the standard criterion based on estimating both speed and length of passing vehicles with two loops and with other methods in the literature.

We are working on incorporating new types of vehicles, such as buses or motorbikes, to the classification algorithm and on researching other supplementary techniques that could improve the success rates of the classification of vans and trucks. We are also developing more sophisticated models of the ILD model that will allow us to obtain more accurate vehicle profiles in order to improve the simulator.

## Figures and Tables

**Figure A1:**
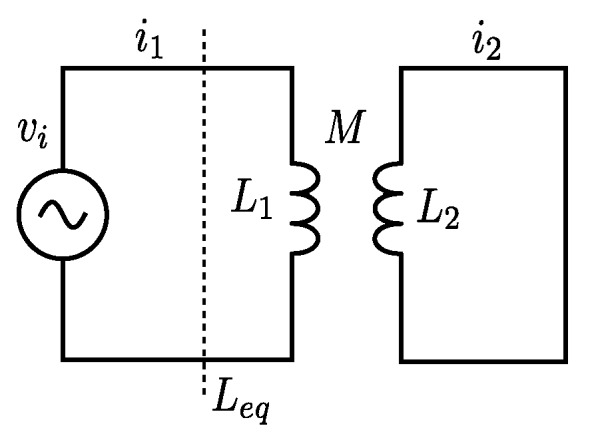
Equivalent circuit coil-vehicle undercarriage.

**Figure A2:**
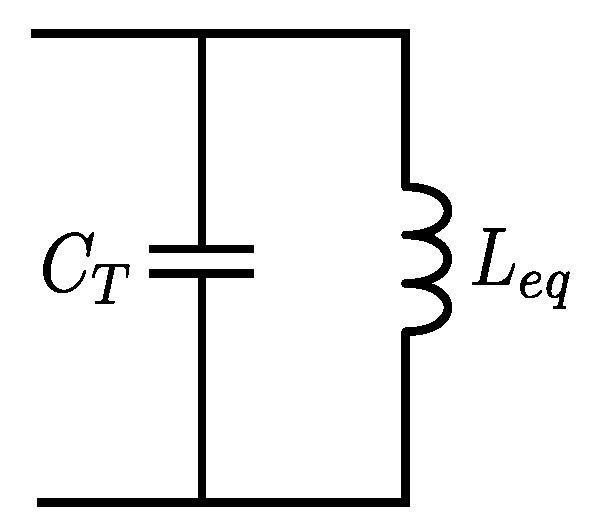
Resonant oscillation circuit used for vehicle detection.

## Appendix

### A. Equivalent Model

The assembly formed by the coil and the vehicle undercarriage is modeled by an air core transformer in which the primary coil inductance, denoted by $L_{1}$, is excited by the sinusoidal generator at the oscillation frequency; the secondary, which represents the vehicle undercarriage, is modeled by a turn in short circuit with an inductance denoted by $L_{2}\left(t\right)$, whose value changes with the vehicle position on the coil located under the road pavement; and finally, the coupling coil-vehicle is modeled by the mutual inductance denoted by M(t), which also depends of the position of the vehicle on the road coil. This equivalent circuit is shown in Figure A1.

From the mesh equations obtained in sinusoidal steady-state

$$\begin{array}{cc} v_{i} & =i_{1}j\omega L_{1}-i_{2}jwM\\ 0 & =-i_{1}j\omega M+i_{2}jwL_{2} \end{array}$$

We determine the input impedance

$$Z_{i}=\frac{v_{i}}{i_{1}}=j\omega \left(\frac{L_{1}L_{2}-M^{2}}{L_{2}}\right)=j\omega L_{eq}$$

which explains Equation (6) of the paper.

Most detectors of inductive loops do not directly measure changes in $L_{eq}$ but changes in period or frequency of a resonant oscillating circuit used for vehicle detection (see Figure A2). The oscillator frequency is controlled by a parallel resonant circuit, also called tank circuit, which consists of the inductance $L_{eq}$ of the loop in parallel with a capacity, denoted by $C_{T}$, located at the detector.

The oscillation frequency is given by

$$f=\frac{1}{T}=\frac{1}{2\pi \sqrt{L_{eq}C_{T}}}$$

and thus the difference in the oscillation period with a vehicle over the loop and without it is

(A1) $$\Delta T=2\pi \left(\sqrt{L_{1}C_{T}}-\sqrt{L_{eq}C_{T}}\right)$$

which justifies Equation (5).

### B. DFT Descriptor Properties

In this Appendix, we demonstrate the independence of the proposed descriptor on the vehicle speed and on the lateral displacement over the inductive loop.

#### B.1. Independence on Speed

Let r(t) be the inductive signature obtained for a constant vehicle speed *v*, which corresponds to a Fourier transform r[ω]. Thus, the DFT descriptor proposed in the paper is given by

$$R\left[\omega_{n}\right]=\frac{\left|r[\omega_{n}]\right|}{\left|r[\omega_{0}]\right|}$$

Let $r(t_{i})$ the inductive signature in the time instant $t=t_{i}$ and $d=vt_{i}$ the distance traveled by the vehicle. For a vehicle speed $v^{\prime }=av$, where *a* is a positive real value, the distance traveled by the vehicle is $d^{\prime }=avt_{i}$, which corresponds to a time instant $t^{\prime }=d^{\prime }/v=at_{i}$. Thus, we obtain an inductive signature $r^{\prime }\left(t\right)=r\left(at\right)$, which gives us the Fourier transform $r^{\prime }\left[\omega \right]$. Using the scaling property of the Fourier transform, $r^{\prime }\left[\omega \right]$ is scaled by 1/a in both amplitude and frequency. That means that if a vehicle is traveling with speed *v* and the Fourier transform of its inductive signature has a local maximum at the frequency bin $\omega_{n}$, for a vehicle with speed $v^{\prime }=av$, that maximum appears at the frequency bin $\omega_{n}/a$ and its amplitude is divided by *a*. Thus, we have

$$r^{\prime }\left[\frac{\omega_{n}}{a}\right]=\frac{1}{a}r\left[\omega_{n}\right]$$

Therefore, the DFT descriptor corresponding to a vehicle with speed $v^{\prime }$ is expressed as

$$R^{\prime }\left[\frac{\omega_{n}}{a}\right]=\frac{|r^{\prime }\left[\frac{\omega_{n}}{a}\right]|}{|r^{\prime }\left[\frac{\omega_{0}}{a}\right]|}=\frac{|ar^{\prime }\left[\frac{\omega_{n}}{a}\right]|}{|ar^{\prime }\left[\frac{\omega_{0}}{a}\right]|}=\frac{\left|r[\omega_{n}]\right|}{\left|r[\omega_{0}]\right|}=R\left[\omega_{n}\right]$$

#### B.2. Independence on Lateral Displacement

Considering now that a lateral displacement can cause a partial coverage of the vehicle on the coil, we will see how our descriptor responds to variations in lateral displacement using our physical model. Let *b* be the vehicle coverage or its effective width on the coil, which is less than or equal to the coil width, denoted by *a*. Suppose that the vehicle is completely on the coil and that produces the maximum amplitude of inductive signature ΔT. Thus, we have

$$\begin{array}{c} L_{1}=\frac{\mu_{0}N_{1}^{2}a^{2}F_{1}}{l_{1}}\\ L_{2}\left(b\right)=\frac{\mu_{0}abF_{2}}{l_{2}}\\ M\left(b\right)=\frac{\mu_{0}N_{1}abF_{1}}{d(b)}\\ L_{eq}\left(b\right)=L_{1}-\frac{M^{2}\left(b\right)}{L_{2}\left(b\right)}=L_{1}-\frac{\mu_{0}N_{1}^{2}aF_{1}^{2}l_{2}}{F_{2}d^{2}}b\\ \Delta T=2\pi \left(\sqrt{L_{1}C_{T}}-\sqrt{L_{eq}\left(b\right)C_{T}}\right)=2\pi \left(\sqrt{L_{1}C_{T}}-\sqrt{\left(L_{1}-\frac{\mu_{0}N_{1}^{2}aF_{1}^{2}l_{2}}{F_{2}d^{2}}b\right)C_{T}}\right)\\ \;\;=2\pi \left(\sqrt{L_{1}C_{T}}-\sqrt{L_{1}C_{T}\left(1-\frac{l_{1}l_{2}F_{1}}{aF_{2}d^{2}}b\right)}\right)=2\pi \sqrt{L_{1}C_{T}}\left(1-\sqrt{1-\frac{l_{1}l_{2}F_{1}}{aF_{2}d^{2}}b}\right) \end{array}$$

where $\mu_{0}=4\pi \times 10^{-7}\;\text{H/m}$, $N_{1}$ is the number of turns, $a^{2}$ is the cross sectional area of the coil, $l_{1}$ is the axial length of the coil, $F_{1}$ is a factor used to consider the non uniform flux in the roadway inductive loop, $l_{2}$ is the axial length of the loop, and $F_{2}$ is the same factor as $F_{1}$ but referred to the vehicle inductive loop. The average distance from the road loop to the vehicle undercarriage is denoted by *d*. Only one turn is considered in our model, *i.e.*, $N_{2}=1$.

Since $l_{1}l_{2}F_{1}/aF_{2}d^{2}<<1$, we can make an approximation of the square root by the first two terms of the Taylor series as follows

(B1) $$\Delta T≅2\pi \sqrt{L_{1}C_{T}}\left(1-\left(1-\frac{l_{1}l_{2}F_{1}}{2aF_{2}d^{2}}b\right)\right)=\pi \sqrt{L_{1}C_{T}}\frac{l_{1}l_{2}F_{1}}{aF_{2}d^{2}}b$$

Let r(t) be the vehicle inductive signature of a vehicle with effective width *b*. For an effective width $b^{\prime }=cb$, where *c* is a positive real value, we obtain an inductive signature $r^{\prime }\left(t\right)=cr\left(t\right)$, since each point ΔT conforming the signature is linearly dependent on *b* as shown in Equation (B1). The Fourier transform of this inductive signature, denoted as $r^{\prime }\left[\omega \right]$, is thus given by

$$r^{\prime }\left[\omega \right]=cr\left[\omega \right]$$

due to the linearity of the DFT. The corresponding descriptor is then given by

$$R^{\prime }\left[\omega_{n}\right]=\frac{|r^{\prime }\left[\omega_{n}\right]|}{|r^{\prime }\left[\omega_{0}\right]|}=\frac{\left|cr[\omega_{n}]\right|}{\left|cr[\omega_{0}]\right|}=\frac{\left|r[\omega_{n}]\right|}{\left|r[\omega_{0}]\right|}=R\left[\omega_{n}\right]$$
